# The impact of metabolic syndrome and triglyceride-glucose index on the risk of in-stent restenosis after percutaneous coronary intervention: a retrospective study

**DOI:** 10.3389/fcvm.2026.1780840

**Published:** 2026-04-10

**Authors:** Lin Han, Yanchun Chen, Liang Xu, Wanhua Chen

**Affiliations:** Department of Cardiovascular Medicine, Yixing People’s Hospital, Yixing, China

**Keywords:** COX analysis, in-stent restenosis, logistic regression, metabolic syndrome, ROC curve, TyG index

## Abstract

**Background:**

In-stent restenosis (ISR) remains a significant clinical challenge after percutaneous coronary intervention (PCI). Metabolic syndrome (MetS), characterized by a cluster of metabolic abnormalities, is increasingly recognized as a contributor to cardiovascular disease progression. This study aimed to investigate the association between MetS and the risk of ISR.

**Methods:**

We retrospectively reviewed the clinical and laboratory data of patients who underwent PCI and had follow-up angiography. Patients were categorized into MetS and non-MetS groups. MetS for comparisons of clinical characteristics and metabolic parameters. Cox and logistic regression analysis was used to identify independent risk factors for ISR. The predictive value of the triglyceride-glucose (TyG) index was evaluated using receiver operating characteristic (ROC) curve analysis.

**Results:**

A total of 565 patients were included, of whom 99 (17.5%) developed ISR and of whom 146 (25.8%) developed MetS. The prevalence of MetS was significantly higher in the ISR group than in the non-ISR group (46.5% vs. 21.5%). Patients with MetS had significantly higher fasting plasma glucose, triglyceride, TyG index, BMI, and blood pressure, but reduced high-density lipoprotein cholesterol. Multivariable logistic regression revealed MetS (OR = 2.43, 95% CI: 1.43–4.15) and TyG index (OR = 2.65, 95% CI: 1.55–4.52) as independent predictors of ISR. ROC analysis demonstrated that the TyG index had good discriminatory power for ISR (AUC = 0.70).

**Conclusion:**

Metabolic syndrome is significantly associated with an increased risk of ISR following PCI. The TyG index may serve as a useful marker for predicting ISR in clinical practice.

## Introduction

Percutaneous coronary intervention (PCI) has revolutionized the treatment of coronary artery disease (CAD), significantly reducing the incidence of acute coronary syndromes and improving patient outcomes (1, 2). However, despite the advent of drug-eluting stents (DES), in-stent restenosis (ISR) remains a notable clinical challenge, occurring in approximately 5%–20% of patients depending on risk factors and stent types (3, 4). ISR, defined as the re-narrowing of the stented artery segment due to neointimal hyperplasia, may lead to recurrent angina, repeat revascularization, and adverse cardiovascular events, imposing a considerable burden on patients and healthcare systems (5).

In recent years, growing attention has been directed toward metabolic syndrome (MetS) as a potential contributor to adverse cardiovascular outcomes, including ISR. MetS is a cluster of interrelated metabolic abnormalities, encompassing central obesity, hypertension, dyslipidemia, and hyperglycemia (6, 7). It affects nearly 25% of the global adult population and is strongly associated with atherogenesis, endothelial dysfunction, and systemic inflammation—pathophysiological processes that also underlie restenosis (8–10). Several studies have demonstrated that individuals with MetS are at higher risk for both the development and progression of CAD (7–9). However, its direct association with ISR remains incompletely understood and has yielded inconsistent findings in the literature.

Moreover, the clinical utility of composite metabolic indices, such as the triglyceride-glucose (TyG) index, has garnered increasing interest. The TyG index, derived from fasting triglyceride and glucose levels, has emerged as a surrogate marker of insulin resistance—a central mechanism in MetS (11–13)—and has been linked to subclinical atherosclerosis, arterial stiffness, and adverse cardiovascular outcomes (14, 15). Unlike the formal MetS criteria, which rely on categorical cutoffs, the TyG index provides a continuous and quantifiable metabolic risk estimate that may better capture subtle variations in metabolic status. Its role in predicting ISR, however, remains underexplored.

Given these considerations, we conducted a retrospective cohort study to assess the relationship between MetS and ISR in patients undergoing PCI and to evaluate the predictive value of the TyG index. We further examined the contribution of individual MetS components and oxidative stress markers to ISR risk. By integrating metabolic and inflammatory parameters, this study aims to provide a more comprehensive understanding of the factors contributing to restenosis and to identify potential markers for ISR risk stratification in clinical practice.

## Materials and methods

### Study design and population

We conducted a retrospective cohort study involving patients who underwent successful PCI with follow-up coronary angiography between 2015 and 2024 at Yixing People's Hospital. ISR was defined as angiographically confirmed restenosis detected during follow-up after hospital discharge. Acute or hyperacute stent thrombosis occurring during the hospitalization was not classified as ISR. Cases of early procedural complications, including acute stent thrombosis potentially related to stent malapposition at the time of implantation, were not included in the ISR analysis. Patients with incomplete data or acute myocardial infarction within 30 days were excluded. Patients were excluded from the study based on the following criteria: Age <18 years; History of coronary artery bypass grafting (CABG); Culprit lesion treated with bare-metal stents (BMS) or balloon angioplasty without drug-eluting stent (DES) implantation; Suspected familial hypertriglyceridemia (triglyceride level ≥ 5.65 mmol/L); Severe hepatic or renal impairment, defined as an estimated glomerular filtration rate (eGFR) < 30 mL/min/1.73 m²; Presence of acute or chronic inflammatory disease, active malignancy, or morbid obesity (BMI ≥ 45 kg/m²). Patients who underwent a repeat coronary angiography during the follow-up period were included. The indication and timing of the repeated coronary angiography were based on the clinical judgement during routine follow-up without a pre-defined time restriction.

### Data collection

Demographic, clinical, and laboratory data were retrospectively collected from the electronic medical records by trained physicians blinded to the study objectives. The definition of metabolic syndrome (MetS) followed established criteria, such as those proposed by the National Cholesterol Education Program Adult Treatment Panel III (NCEP ATP III) or the International Diabetes Federation (IDF).

The triglyceride-glucose (TyG) index was calculated using the formula:

Ln [fasting triglycerides (mg/dL) × fasting glucose (mg/dL)/2].

Clinical data included patient age, sex, smoking status, comorbid conditions, left ventricular ejection fraction (LVEF), coronary angiographic findings, procedural details, and discharge medications.

Peripheral venous blood samples were drawn after an overnight fast of at least 8 h and analyzed in the central laboratory of Beijing Anzhen Hospital, Capital Medical University. Laboratory measurements included fasting blood glucose (FBG), uric acid, estimated glomerular filtration rate (eGFR), high-sensitivity C-reactive protein (hs-CRP), and lipid profile parameters: triglycerides (TG), total cholesterol (TC), low-density lipoprotein cholesterol (LDL-C), and high-density lipoprotein cholesterol (HDL-C).

The TG and FBG were originally reported in unit of mmol/L. The concentration units were converted into mg/dL following the conversion factor: TG (1 mmol/L = 88.5 mg/dL) and FBG (1 mmol/L = 18 mg/dL). The triglyceride-glucose (TyG) index was calculated using the formula:

Ln [fasting triglycerides (mg/dL) × fasting glucose (mg/dL)/2].

Body mass index (BMI) was calculated as weight in kilograms divided by height in meters squared (kg/m²).

Smoking status was classified as never, former (cessation >1 month), or current smoker.

Hypercholesterolemia was defined as TC > 6.22 mmol/L, LDL-C > 4.14 mmol/L, or current use of lipid-lowering medications, according to the 2018 AHA/ACC guideline (16).

Diabetes mellitus (DM) was diagnosed based on a documented medical history, the use of antidiabetic therapy, or classical hyperglycemic symptoms accompanied by FBG > 7.0 mmol/L and/or random plasma glucose >11.1 mmol/L, according to the American Diabetes Association criteria (17).

Based on coronary angiography, multivessel disease was defined as significant stenosis (≥50% luminal narrowing) in two or more major coronary arteries.

Chronic total occlusion (CTO) was defined as a complete blockage of a native coronary artery with an estimated duration of at least 3 months.

Outcome Definition: ISR was defined as ≥50% diameter stenosis within the stent or at its edges based on follow-up angiography.

### Statistical analysis

All statistical analyses were performed using SPSS version 30.0 (IBM Corp., Armonk, NY, USA) and R software version 4.4.1 (R Foundation for Statistical Computing, Vienna, Austria). A two-sided *p*-value of < 0.05 was considered statistically significant.

Baseline characteristics were analyzed to compare patients with and without metabolic syndrome (MetS). The dataset was processed using Python (version 3.x) and the pandas library. Patients were categorized into MetS and non-MetS groups based on the presence of diagnostic criteria. Continuous variables, including age, BMI, fasting plasma glucose (FPG), lipid levels, estimated glomerular filtration rate (eGFR), and left ventricular ejection fraction (LVEF), were summarized as means with standard deviations (mean ± SD). Categorical variables, such as sex, hypertension, diabetes mellitus, and hyperlipidemia, were expressed as frequencies and percentages. No imputation was performed for missing data. This analysis was descriptive in nature and did not include formal hypothesis testing for group comparisons.

Continuous variables were tested for normality using the Kolmogorov–Smirnov test. Variables with a normal distribution were expressed as mean ± standard deviation (SD), and comparisons between groups (ISR vs. non-ISR) were conducted using the independent samples *t*-test. Non-normally distributed variables were presented as median (interquartile range) and compared using the Mann–Whitney *U* test. Categorical variables were expressed as counts and percentages and compared using the Chi-square test or Fisher's exact test, as appropriate.

To identify factors independently associated with in-stent restenosis (ISR), univariate logistic regression was initially performed. Variables with a *p*-value < 0.1 in univariate analysis were entered into a multivariable logistic regression model using a forward stepwise method. Odds ratios (ORs) and 95% confidence intervals (CIs) were calculated to estimate the strength of association.

## Results

### Characteristics of patients

A total of 565 patients who underwent percutaneous coronary intervention (PCI) were included in this study. As shown in Table 1, 146 (25.8%) were diagnosed with metabolic syndrome (MetS) and 99 (17.5%) were diagnosed with ISR. The occurrence of MetS was significantly higher in ISR group than that in non-ISR group (46.5% vs. 21.5%). Furthermore, compared to non-MetS group, MetS group displayed similar age (Non-MetS: 60.5 ± 10.8 vs. MetS: 61.4 ± 10.8 years), male predominance (Non-MetS: 84.9% vs. MetS: 85.0%), low-density lipoprotein cholesterol (LDL-C) (Non-MetS: 1.88 ± 0.59 vs. MetS: 1.90 ± 0.59 mmol/L), estimated glomerular filtration rate (eGFR) (Non-MetS: 85.3 ± 28.9 vs. MetS: 84.2 ± 30.9 mL/min/1.73 m²), and left ventricular ejection fraction (LVEF) (Non-MetS: 58.9 ± 7.3 vs. MetS: 59.1% ± 6.9%). In contrast, patients in the MetS group exhibited a higher body mass index (BMI) than those without MetS (25.4 ± 2.6 vs. 24.4 ± 2.5 kg/m²). The prevalence of hypertension and diabetes mellitus was significantly higher in the MetS group (52.7% vs. 42.1% and 76.0% vs. 17.4%, respectively). In terms of laboratory parameters, the MetS group had elevated fasting plasma glucose (FPG: 102.9 ± 27.1 vs. 137.3 ± 51.6 mg/dL), triglyceride levels (TG: 139.5 ± 72.8 vs. 171.8 ± 81.8 mg/dL), triglyceride-glucose index (TyG) (9.21 ± 0.59 vs. 8.74 ± 0.53), along with reduced high-density lipoprotein cholesterol (HDL-C: 0.93 ± 0.18 vs. 1.11 ± 0.26 mmol/L).

**Table 1:** Characteristics comparison between patients with and without MetS.

Categories		Non-MetS (*n* = 419)	MetS (*n* = 146)	*P* value
ISR	No	366 (64.8%)	100 (17.7%)	<0.001
	Yes	53 (9.4%)	46 (8.1%)	
Age		61.5 ± 10.8	60.5 ± 10.8	0.383
Gender	Female	63 (11.2%)	22 (3.9%)	0.992
	Male	356 (63%)	124 (21.9%)	
BMI (kg/m²)		24.4 ± 2.5	25.4 ± 2.6	<0.001
Hypertension	No	231 (40.9%)	69 (12.2%)	0.101
	Yes	168 (29.7%)	77 (13.6%)	
Diabetes Mellitus	No	346 (61.2%)	35 (6.2%)	<0.001
	Yes	73 (12.9%)	111 (19.6%)	
FPG (mg/dL)		102.9 ± 27.1	137.3 ± 51.6	<0.001
Triglyceride (mg/dL)		139.5 ± 72.8	171.8 ± 81.8	<0.001
TyG		8.74 ± 0.53	9.21 ± 0.59	<0.001
HDL-C (mM)		1.11 ± 0.26	0.93 ± 0.18	<0.001
LDL-C (mM)		1.88 ± 0.59	1.90 ± 0.59	0.726
eGFR (mL/min/1.73m²)		85.3 ± 28.9	84.2 ± 30.9	0.757
LVEF (%)		58.9 ± 7.3	59.1 ± 6.9	0.724

In addition, compared to non-ISR patients, serum from ISR patients contained higher concentrations of MDA (4.24 ± 0.93 vs. 1.90 ± 0.70 μmol/L, *P* < 0.001), but lower GSH (3.01 ± 0.56 vs. 4.84 ± 0.87 μmol/L, *P* < 0.001) and SOD (105.34 ± 16.73 vs. 150.93 ± 22.27 U/mL, *P* < 0.001).

### Association between metabolic syndrome and cardiometabolic risk factors

**Figure 1:**
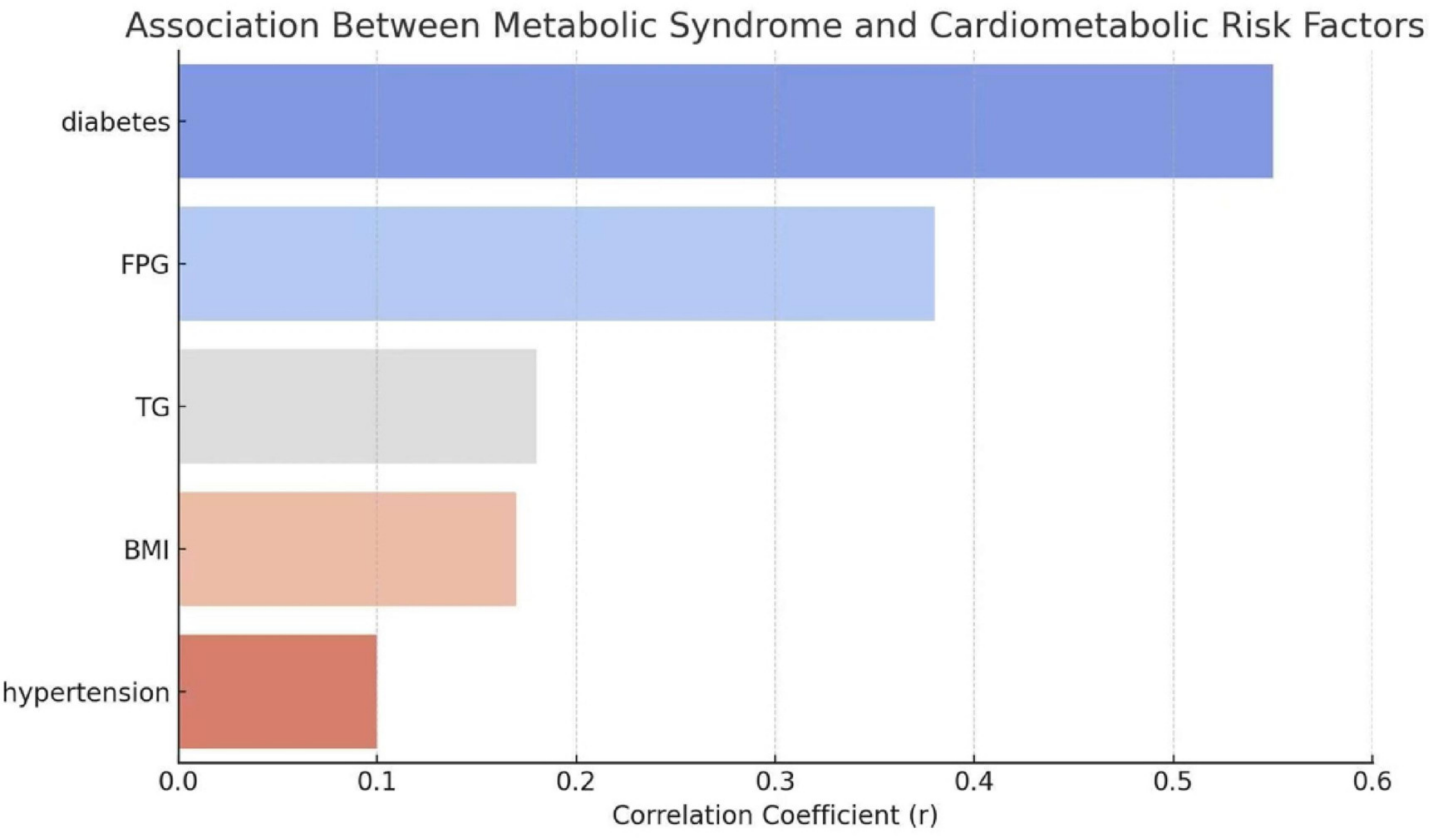
Association between metabolic syndrome and cardiometabolic risk factors. Bar chart illustrating correlation coefficients (r) between metabolic syndrome and major cardiometabolic risk factors, including diabetes mellitus, fasting plasma glucose (FPG), triglycerides (TG), body mass index (BMI), and hypertension. Metabolic syndrome showed a strong positive association with diabetes mellitus and moderate associations with fasting plasma glucose, while weaker correlations were observed with triglyceride levels, body mass index, and hypertension, highlighting the clustered nature of metabolic abnormalities.

To explore the relationship between metabolic syndrome (MetS) and cardiometabolic risk factors, a correlation analysis was performed. As shown in Figure 1, the results revealed that MetS was strongly associated with the presence of diabetes mellitus (*r* = 0.55), indicating a moderate to strong positive correlation. Additionally, fasting plasma glucose (FPG) demonstrated a notable positive association with MetS (*r* = 0.38), reflecting the metabolic component of the syndrome. Triglyceride levels (TG; *r* = 0.18) and body mass index (BMI; *r* = 0.17) also showed weaker yet positive correlations. Hypertension was modestly associated with MetS (*r* = 0.10). These findings highlighted the clustering of multiple metabolic risk factors in patients with MetS, supporting its role as a composite predictor of cardiometabolic burden.

### Association between number of metabolic syndrome components and clinical outcomes

**Figure 2:**
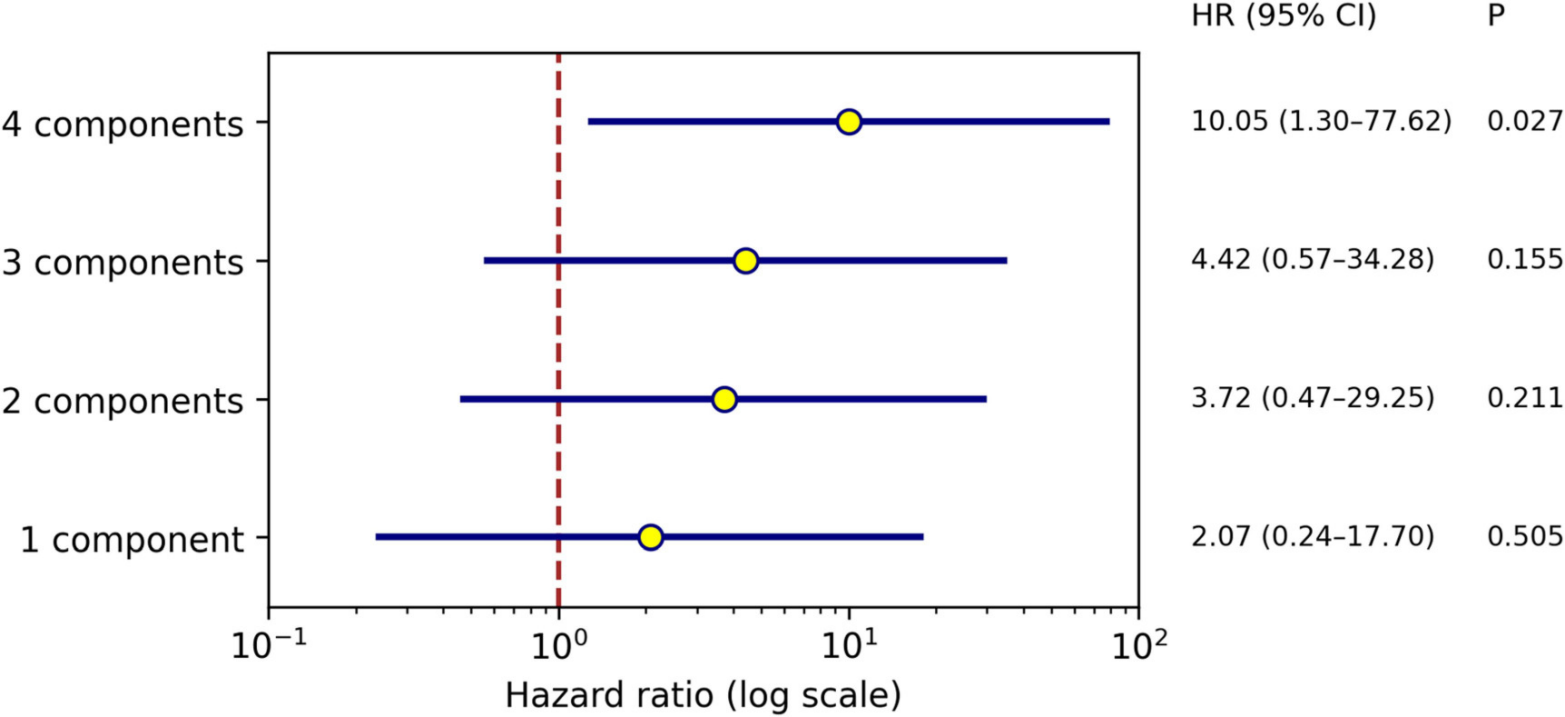
Association between the number of metabolic syndrome components and clinical outcomes. Hazard ratios (HRs) with 95% confidence intervals (CIs) for the combined clinical endpoint according to the number of metabolic syndrome (MetS) components are shown, using patients without MetS components as the reference group. The risk of the combined endpoint increased progressively with a greater number of MetS components, reaching statistical significance in patients with four components (*p* = 0.027), suggesting a dose–response relationship between cumulative metabolic burden and adverse clinical outcomes.

Cox proportional hazards regression analysis was performed to evaluate the association between the number of metabolic syndrome (MetS) components and the risk of experiencing the combined endpoint (Figure 2). Patients with non-MetS components served as the reference group. Compared to this reference, patients with one, two, and three components demonstrated progressively higher, though not statistically significant, odds of the combined endpoint, with hazard ratios (HR) of 2.07 (95% CI: 0.24–17.70; *p* = 0.505), 3.72 (95% CI: 0.47–29.25; *p* = 0.211), and 4.42 (95% CI: 0.57–34.28; *p* = 0.155), respectively. Notably, patients with four MetS components had a significantly increased risk of the combined endpoint, with an HR of 10.05 (95% CI: 1.30–77.62; *p* = 0.027). These findings suggested a dose-dependent relationship between the number of MetS components and adverse cardiovascular outcomes, reinforcing the cumulative risk burden posed by metabolic dysregulation.

### Multivariable logistic regression analysis

**Figure 3:**
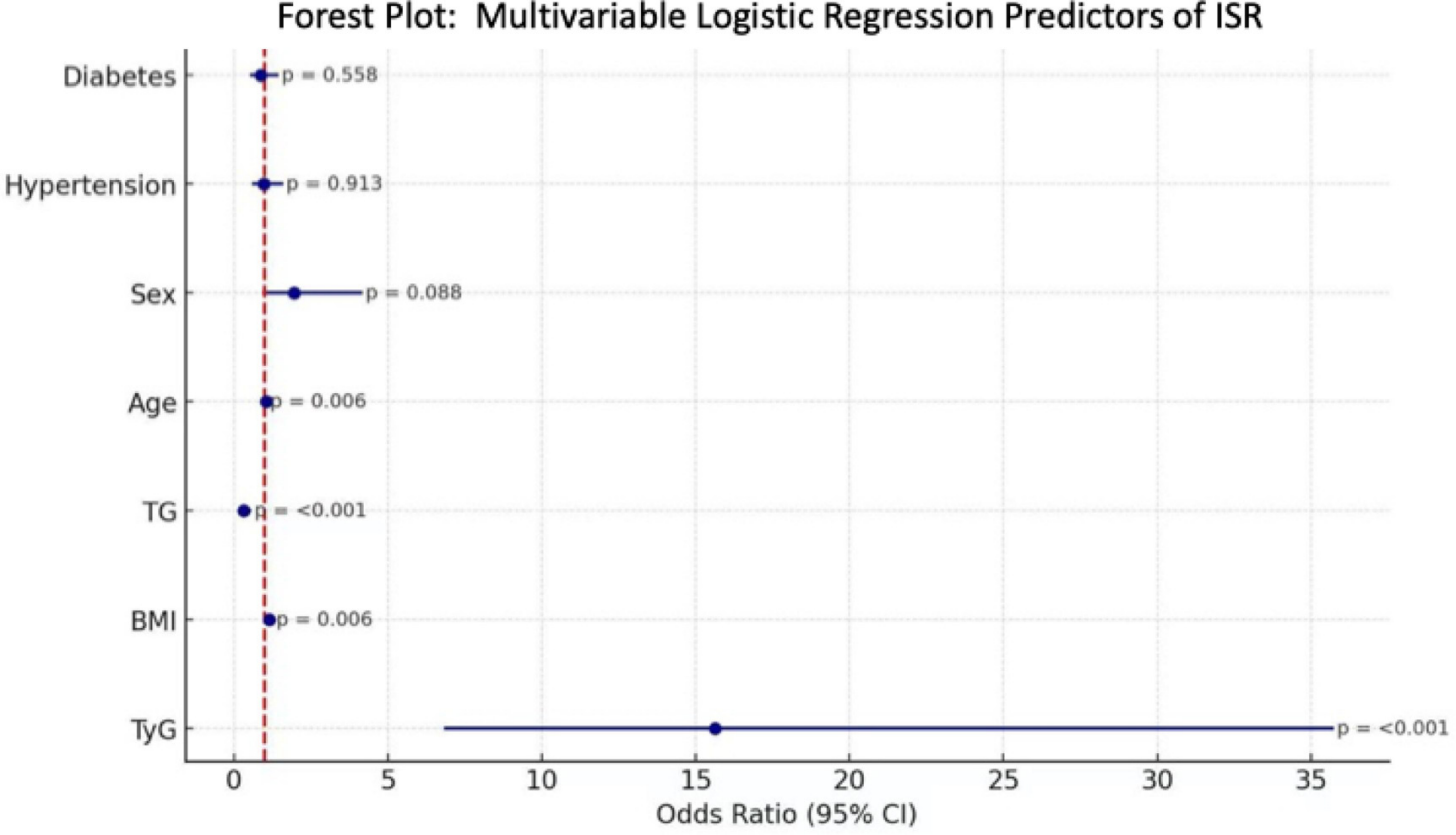
Forest plot of multivariable logistic regression analysis for predictors of in-stent restenosis. Forest plot showing adjusted odds ratios (ORs) with 95% confidence intervals (CIs) for variables independently associated with in-stent restenosis (ISR). The triglyceride–glucose (TyG) index emerged as a strong independent predictor of ISR, while body mass index (BMI) and age were also significantly associated. In contrast, sex, hypertension, diabetes mellitus, and triglyceride levels were not independently associated with ISR after multivariable adjustment. The vertical dashed line indicates the line of no effect (OR = 1).

Multivariable logistic regression was performed to identify the independent predictors of in-stent restenosis (ISR). As shown in Figure 3, the TyG index emerged as a strong independent predictor of ISR, with an odds ratio (OR) significantly greater than 1 (OR = 13.59, 95% CI: 4.96–37.20, *p* < 0.001), indicating a markedly increased risk in patients with elevated TyG index. In addition, traditional metabolic parameters such as BMI and triglyceride levels also demonstrated significant associations. Specifically, BMI was positively associated with ISR (OR = 1.14, 95% CI: 1.04–1.26, *p* = 0.006), while elevated triglyceride levels showed an inverse relationship (OR = 0.32, *p* < 0.001), which might reflect collinearity with the TyG index or confounding.

Age was inversely associated with ISR risk (OR = 0.97, 95% CI: 0.91–0.99, *p* = 0.006), suggesting younger patients might be more prone to restenosis, potentially due to more aggressive neointimal proliferation. Meanwhile, sex (OR = 1.96, 95% CI: 0.91–4.31, *p* = 0.088), hypertension (OR = 1.01, *p* = 0.96), and diabetes mellitus (OR = 0.85, *p* = 0.56) were not significant predictors after multivariable adjustment.

**Figure 4:**
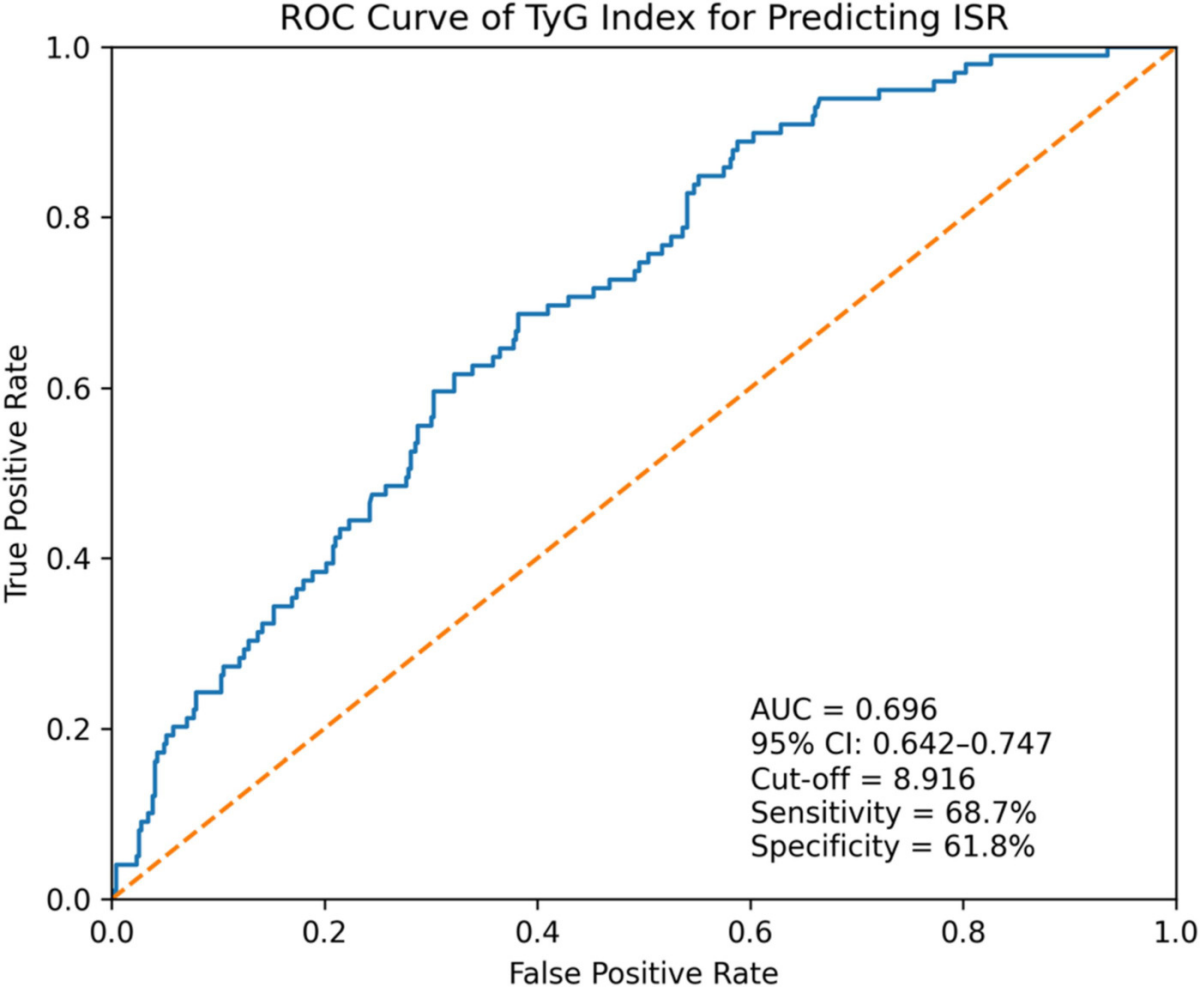
Receiver operating characteristic (ROC) curve of the triglyceride–glucose (TyG) index for predicting in-stent restenosis.

To further evaluate the discriminative performance of TyG index for predicting ISR, a receiver operating characteristic (ROC) curve analysis was performed. As shown in Figure 4, the TyG index demonstrated a moderate ability to distinguish patients with ISR from those without ISR, with an area under the curve (AUC) of 0.70. This result indicated that TyG index had acceptable predictive accuracy for ISR, supporting its role as an independent predictor in this population.

The ROC curve illustrates the discriminative performance of the TyG index for predicting in-stent restenosis (ISR) in patients undergoing percutaneous coronary intervention. The diagonal dashed line represents the reference line of no discrimination. Optimal cut-off value (8.916), sensitivity (68.7%), specificity (61.8%), and 95% confidence interval (CI) of the AUC (0.642–0.747) were obtained in the ROC analysis.

## Discussion

In this retrospective cohort study, we demonstrated that metabolic syndrome (MetS) is significantly associated with an increased risk of in-stent restenosis (ISR) following percutaneous coronary intervention (PCI). Among the metabolic parameters evaluated, the triglyceride-glucose (TyG) index emerged as a particularly strong and independent predictor. These findings highlighted the substantial influence of metabolic dysregulation on vascular healing and reinforce the importance of comprehensive metabolic assessment in patients undergoing PCI.

Our results are consistent with prior studies that have reported a link between MetS and ISR. For example, previous investigations have shown that insulin resistance and dyslipidemia—core features of MetS—are associated with adverse vascular remodeling and neointimal hyperplasia, both of which are central to ISR pathogenesis (18, 19). The strong correlation we observed between MetS and diabetes mellitus (*r* = 0.55), as well as elevated fasting plasma glucose (FPG), aligns with this mechanistic pathway. Hyperglycemia promotes endothelial dysfunction, increases oxidative stress, and enhances vascular smooth muscle cell proliferation, all of which contribute to restenosis (20).

In addition to confirming known associations, our study provided novel insights by demonstrating a clear dose-response relationship between the number of MetS components and the risk of ISR. Patients with four MetS components had a significantly elevated risk compared to those without any components, suggesting that even partial metabolic derangements can substantially impact vascular outcomes. This finding supported the concept that MetS is not merely a diagnostic label but a continuum of metabolic stress that should be addressed proactively.

The TyG index, calculated from fasting glucose and triglyceride levels, has gained recognition as a surrogate marker of insulin resistance and systemic metabolic burden (14, 21, 22). In our cohort, a higher TyG index was independently associated with ISR, and ROC analysis yielded an AUC of 0.696, indicating good predictive value. The utility of the TyG index lies in its simplicity and availability, as it can be readily calculated from routine blood tests without the need for specialized assays like HOMA-IR (Homeostatic Model Assessment of Insulin Resistance). This makes it particularly valuable for risk stratification in resource-limited settings or for long-term outpatient monitoring. Of note, in the multivariable logistic regression analysis, variable selection was not exclusively based on univariable statistical significance. Sex and hypertension were selected considering their well-recognized role as cardiovascular risk factors and potential confounders. This emphasizes adjustment for clinically relevant variables rather than reliance solely on *p*-value–driven selection.

Interestingly, we also found that ISR patients had significantly higher levels of malondialdehyde (MDA) and reduced levels of antioxidant enzymes such as superoxide dismutase (SOD) and glutathione (GSH), indicating an imbalance in redox homeostasis. Oxidative stress is a well-established contributor to ISR, mediating endothelial injury, inflammation, and extracellular matrix remodeling (23, 24). The integration of oxidative stress markers into future predictive models may further improve ISR risk assessment.

Beyond biological mechanisms, our findings may provide potential clinical relevance. The identification of MetS and TyG index as predictors of ISR suggests that metabolic risk could identify patients at higher risk following PCI. However, whether aggressive management of metabolic risk factors—particularly glucose and lipid abnormalities—can play a role in reducing post-PCI complications is still required for further determination.

Moreover, our study adds to the limited body of literature focused on east Asian populations, who may exhibit distinct metabolic profiles compared to western cohorts. For example, Asian patients often develop insulin resistance and central obesity at lower BMI thresholds, which may contribute to under-recognition of metabolic risk (25–29). The predictive utility of the TyG index in this context reinforces its applicability across diverse populations and suggests it may capture pathophysiological processes not fully reflected by traditional MetS criteria (10, 30–33).

Nonetheless, several limitations merit discussion. First, the retrospective and single-center nature of our study may introduce selection and information bias. Second, while we adjusted for major confounders, residual confounding cannot be ruled out. Third, ISR was defined angiographically rather than clinically, and therefore may not reflect symptomatic restenosis. Fourth, the cross-sectional assessment of metabolic parameters precludes evaluation of temporal changes or treatment effects. Fifth, the relatively modest sample size may have limited the power to detect associations with less common MetS components or rare outcomes. Sixth, the decision to perform repeat angiography was based on clinical judgement during follow-up with not pre-defined time restriction. However, detailed categorization of the specific clinical indications was not consistently available, which may introduce potential selection bias. Finally, although most patients received standard antiplatelet therapy (aspirin and/or clopidogrel) following PCI in accordance with guideline recommendations, detailed data regarding long-term adherence during follow-up could not be verified, which may represent a potential confounding factor. Finally, operator experience and skills could not be standardized or adjusted for, and these factors may have influenced ISR outcomes.

Future research should focus on prospective validation of our findings in multicenter cohorts, ideally incorporating serial measurements of metabolic parameters and integrating clinical outcomes such as target lesion revascularization and major adverse cardiovascular events (MACE). Randomized trials evaluating whether reducing TyG index can reduce ISR rates would also be of significant value.

## Conclusion

Metabolic syndrome and TyG index are both associated with the occurrence of in-stent restenosis (ISR). In patients undergoing percutaneous coronary intervention (PCI), the presence of metabolic syndrome and a high TyG index may warrant closer clinical follow-up. Considering these two variables in patients treated with PCI may contribute to improved patient management.

## Data Availability

The raw data supporting the conclusions of this article will be made available by the authors, without undue reservation.
